# Septic Arthritis After Anterior Cruciate Ligament Reconstruction: A Comparative Study of Pediatric, Adolescent, and Young Adult Populations

**DOI:** 10.1177/23259671251334591

**Published:** 2025-06-09

**Authors:** Deepak Chona, Jeffrey Kay, Nazgol Tavabi, Michael Quinn, Dennis Kramer, Yi-Meng Yen, Melissa Ann Christino, Matthew Milewski, Mininder S. Kocher, James Pruneski, Ata Kiapour, Benton E. Heyworth

**Affiliations:** * Boston Children’s Hospital, Boston, Massachusetts, USA; Investigation performed at Boston Children’s Hospital, Harvard Medical School, Boston, Massachusetts, USA

**Keywords:** ACL, septic arthritis, pediatric, adolescent

## Abstract

**Background:**

Limited evidence exists regarding septic arthritis (SA) after anterior cruciate ligament reconstruction (ACLR) in pediatric and adolescent patients.

**Purpose/Hypothesis:**

The purpose of this study was to compare post-ACLR SA cases during a 21-year period in pediatric and adolescent patients to those in a young adult control cohort. It was hypothesized that the incidence would be similarly low in both subpopulations.

**Study Design:**

Case series; Level of evidence, 4.

**Methods:**

Records of patients who underwent ACLR between 2000 and 2020 were retrospectively reviewed for diagnoses of postoperative SA of the ipsilateral knee, defined by culture-positive arthrocentesis or synovial fluid white blood cell count >50,000 cells/mL. Surgical details, culture results, and clinical course were analyzed. Age-based cohorts—pediatric (<13 years of age), adolescent (13-19 years of age), and young adult (20-35 years of age)—were compared using chi-square tests for age, graft source, graft type, concurrent meniscal procedures, and SA incidence. Time between ACLR and subsequent surgeries was analyzed utilizing *t* tests.

**Results:**

Of 5638 ACLR cases (pediatric: n = 606, 10.7%; adolescent: n = 4123, 73.1%; young adult: n = 909, 16.1%), SA was diagnosed in 13 patients (0.23%; adolescent: n = 12, 0.30%; young adult: n = 1, 0.11%) a median of 15 days (range 6-632 days) after ACLR. One or more arthroscopic irrigation and debridement (I&Ds) (mean, 2; range, 1-3) were performed, and anterior cruciate ligament (ACL) grafts were retained for all patients. The mean clinical follow-up was 3.5 years (range, 0.6-9.2 years). Subsequent surgeries include 2 lysis of adhesions, 3 meniscectomies, 1 meniscal repair, 3 patellar chondroplasties, 1 loose-body removal, 2 patellar biopsies, 1 debridement, and 1 revision ACLR 6 years postoperatively for acute ACL graft rupture. The most common microbes were *Staphylococcus aureus* (n = 3; 23.1%) and other forms of *Staphylococcus* species (n = 9; 69.2%). No significant associations were identified between age-based cohort and graft source, graft type, age, or concurrent meniscal surgery.

**Conclusion:**

Post-ACLR SA was similarly rare for adolescents and young adults, and no cases were identified in 606 pediatric patients. No associations were identified between SA and demographic factors, graft source, graft type, or concurrent meniscal surgery. *Staphylococcus* species were identified in most cases. Aggressive initial surgical treatment with multiple I&Ds was associated with graft retention, with no disproportionate subsequent graft rupture risk.

Septic arthritis after anterior cruciate ligament reconstruction (ACLR) represents a rare complication, with most previous studies demonstrating rates of 0.2% to 1%.^5,6,8,10,19,41,42^ Treatment typically consists of at least 1 arthroscopic irrigation and debridement (I&D) surgery and, in some cases, graft debridement, which generally requires subsequent revision ACLR at a delayed time point.^15,22,23,31,34,39,48^ It is therefore critical that orthopaedic surgeons be aware of the potential for this devastating complication and its possible risk factors.^34,37^

In recent years, multiple studies have investigated the effect of graft type on septic arthritis risk, with 2 studies demonstrating greater risk with hamstring tendon autograft than other common graft options.^6,13,24,30,38,46^ Other studies have compared outcomes after various treatment strategies for the infection, and several have investigated the long-term effect on knee cartilage health after post-ACLR septic arthritis.^1,7,14,26,35^ While most literature supports graft-retaining strategies, magnetic resonance imaging evidence suggesting decreased chondral health and early osteoarthritis highlights the importance of appropriately treating this complication when it does occur.^22,25,31^

However, the focus of most of the existing literature has been on adult patients, despite adolescents emerging as the age group most affected by anterior cruciate ligament (ACL) tears, based on multiple epidemiological studies, and pediatric patients representing the subpopulation with the greatest increase in incidence over the past 2 decades.^28,33^ Thus, a critical void exists in the knowledge base for both pediatric and adolescent subpopulations and their incidence and features of cases of septic arthritis after ACLR. Without this knowledge, it makes accurate counseling of patients difficult, if not impossible. It was therefore the aim of the current study to contribute to this knowledge base by investigating a comprehensive database of all ACLRs performed at a single pediatric referral center during a 21-year period to identify risk factors and evaluate treatment strategies utilized for diagnosed cases of septic arthritis after ACLR in younger patients. A large young adult cohort served as a control population for the 2 pediatric-based age cohorts. We hypothesized that septic arthritis rates in pediatric and adolescent cohorts would be similarly low to those of the young adult control group.

## Methods

A comprehensive registry of all ACLRs performed at a single regional pediatric referral center from January 2000 to December 2020 was queried to identify all cases of septic arthritis after ACLR, with a goal of a minimum of 2 years between the potential detection of this complication and any further follow-up or treatment that an affected patient might undergo. The registry was developed using robust and validated natural language processing (NLP) pipelines, capable of identifying ACL surgery cases and relevant information from preoperative, operative, and postoperative clinical notes.^32,43^ For this study, rule-based NLP models were used to identify potential cases suspicious of septic arthritis using the following seed terms—infection, septic arthritis, I&D, irrigation, debridement, incision, and drainage—and their derivatives. The NLP pipeline was validated on a total of 1000 manually annotated operative and clinical visit notes (mean accuracy, 0.98 ± 0.02).

The chart of each patient identified by the search process was reviewed for potential inclusion in the present study. Patients were included who (1) underwent primary or revision ACLR at this institution and (2) subsequently were diagnosed with septic arthritis of the ipsilateral knee at any point in the postoperative period, which was defined by synovial fluid yielding either positive bacterial cultures or white blood cell count >50,000 cells/mL in the setting of a negative serum Lyme antibody test. Patients who were >3 months postoperative or had a secondary procedure before the development of septic arthritis were included because the principles used to achieve graft retention and infection eradication in this subset can provide potentially valuable insight to treatment algorithms.^45^

Patients were excluded who had undergone index ACLR at other institutions with subsequent treatment at the present institution. Age-based cohorts were established, which included pediatric (<13 years of age), adolescent (13-19 years of age), and young adult (20-35 years of age).^11^

Surgical details from the ACLR, arthroscopic irrigation and debridement (I&D), and any subsequent ipsilateral knee surgeries before or after the I&D were collected. The study period predated the incorporation of lateral extra-articular augmentation procedures and vancomycin preloading of grafts at this institution. Bacterial culture results, clinical course, and clinical outcomes, including subsequent graft debridement, graft tear, or other complications, were collected.

Four cases were included of patients who underwent an additional ipsilateral knee procedure before the development of septic arthritis. Although it was presumed that the septic arthritis in these 4 cases developed due to the subsequent procedure, rather than the ACLR procedure, the senior authors (B.E.H., A.K.) reached consensus that these 4 patients should be included, so as not to underestimate or underreport the true incidence of septic arthritis after ACLR in the age groups studied. In addition, because data from these 4 cases may serve to help understand the principles of treatment of septic arthritis, and related strategies designed to increase the chances of graft retention, or risks of failure to retain the graft that might be specific to these cases, it was thought inclusion was a more methodologically rigorous approach.

Data that were extracted from each patient chart included patient age, sex, time from ACLR to diagnosis and initiation of treatment of septic arthritis, graft source (allograft or autograft), graft type (hamstring, quadriceps, patellar tendon, or iliotibial band), presence and type of concurrent meniscal surgery, case duration, number of I&D procedures, number of lysis of adhesion procedures, number of total subsequent ipsilateral knee operations, bacterial culture results, and antibiotic treatment regimens and duration.

### Statistical Analysis

The incidence of septic arthritis was calculated for each group from the overall denominator of ACLR cases in each age-based cohort contained in the comprehensive database. Chi-square tests were performed to identify associations between the collected variables and the diagnosis of septic arthritis post-ACLR. Student *t* tests were performed to compare case durations between the ACLR procedures in cases that developed septic arthritis and those that did not. A *P* value of .05 was determined as the threshold for statistical significance.

## Results

Between January 2000 and December 2020, the comprehensive institutional ACLR database included 5638 ACLRs performed by 8 different sports medicine fellowship-trained orthopaedic surgeons (D.K., Y.M.Y., M.A.C., M.M., M.S.K., B.E.H.). The clinical follow-up performed for these patients was a mean of 3.5 years (range, 7 months–9.2 years).

Thirteen cases (0.23%) of post-ACLR septic arthritis were identified at a median of 15 days (range, 6-632 days) after ACLR. Four patients were diagnosed with septic arthritis at a mean of 11 months after ACLR, but all 4 had undergone additional ipsilateral knee arthroscopy for the treatment of non–ACL-related conditions within 17 days or fewer preceding the diagnosis of septic arthritis. One patient had undergone arthroscopic plica excision for a complaint of mechanical symptoms, 1 underwent arthroscopic meniscal repair for a new meniscal tear, 1 underwent arthroscopic partial meniscectomy for a meniscal retear, and 1 underwent arthroscopic partial meniscectomy for a new meniscal tear. Excluding these patients, the diagnosis of the remaining 9 cases (0.16%) occurred at a mean of 13.8 days (range, 6-35 days) after ACLR. Relevant demographic and index surgical characteristics of the 13 cases are summarized in Table 1.

**Table 1:** Demographic and Surgical Features of Index ACLR in Post-ACLR Septic Arthritis Cases (n = 13)^*a*^

	Value
Mean age (range)	17.3 y (14-27 y)
Mean follow-up (range)	3.5 y (7 mo–9.2 y)
Sex, n (%)	
Male	7 (53.8)
Female	6 (46.2)
Graft source, n (%)	
Autograft	13 (100)
Allograft	0 (0)
Graft type, n (%)	
Hamstring tendon	11 (84.6)
Patellar tendon	1 (7.7)
Quadriceps tendon	1 (7.7)

a ACLR, anterior cruciate ligament reconstruction.

### Diagnosis

Eight of the 10 patients (80%) for whom temperature was documented at the time of presentation for postoperative knee pain had fevers. All patients underwent knee arthrocentesis, with 4 (30.8%) occurring in the clinic, 7 (53.8%) in the emergency department, and 2 (15.4%) undocumented. Diagnostic results including synovial fluid cell count and serum laboratory values in the patients for whom this information was available are summarized in Table 2.

**Table 2:** Diagnostic Test Results

Test	Median (range)	No. of Patients
Synovial fluid cell count, cells/µL	58,000 (35,000-140,000)	9
Serum white blood cell count, cells/mL	11.78 (6.76-15)	9
Erythrocyte sedimentation rate, mm/h	75 (37-101)	9
C-reactive protein, mg/L	11.23 (6.77-23.17)	9
Platelet count, cells/µL	381 (230-454)	9

All patients demonstrated positive bacterial cultures, most commonly *Staphylococcus aureus* (n = 3; 23.1%) and other *Staphylococcus* species (n = 9; 69.2%). One patient was positive for both *Staphylococcus hominis* and *Cutibacterium acnes*. Culture results are summarized in Table 3.

**Table 3:** Bacterial Culture Results

Organism	No. of Cases (%)
*S. aureus*	3 (23.1)
Other *Staphylococcus* species^*a*^	9 (69.2)
*Streptococcus* ^*b*^	1 (7.7)
*C. acnes*	1 (7.7)

a Including *Staphylococcus hominis*.

b Group B Strep.

### Treatment

All patients underwent at least 1 arthroscopic I&D, with a mean of 2 procedures (range, 1-3 procedures), and received intravenous antibiotics while in the inpatient setting. Nine patients (69.2%) were discharged on intravenous antibiotics after having received a peripherally inserted central catheter line during their inpatient stay, while 4 (30.8%) were discharged with prescriptions for oral antibiotics. The mean duration of antibiotic therapy was 6 weeks (range, 3-7 weeks). A strategy of attempted graft retention was pursued in all patients. Relevant treatment characteristics are summarized in Table 4.

**Table 4:** Septic Arthritis Treatment Characteristics^*a*^

	Value
Median time, ACLR to I&D (range)^*b*^	15 days (6-632 days)
Median time, ACLR to I&D (range)^*c*^	8 mo (7-21 mo)
Mean no. of I&Ds (range)	2 (1-3)
Postdischarge antibiotic treatment, n (%)	
IV	9 (69.2)
PO	4 (30.8)
Mean antibiotic duration (range)	6 wk (3-7 wk)

a ACLR, anterior cruciate ligament reconstruction; I&D, irrigation and debridement; IV, intravenous; PO, by mouth.

b Patients who were diagnosed with septic arthritis after ACLR without additional surgical procedures before diagnosis.

c Patients who underwent subsequent surgical procedures after ACLR before diagnosis of septic arthritis.

Data were grouped by graft source, graft type, patient age, and presence of concurrent meniscal surgery at the time of index ACLR. Results by graft source, graft type, age, and presence of concurrent meniscal surgery are shown in Tables 5, 6, 7, and 8, respectively. No statistically significant associations were identified by chi-square testing with any of these variables.

**Table 5:** Chi-Square Results of Septic Arthritis Post-ACLR by Graft Source^*a*^

Graft Source	Total	Septic Arthritis Cases, n (%)
Autograft (incidence)	5268	13 (0.25)
Allograft	368	0 (0)
Degrees of freedom	3	
*P* value	.28	

a ACLR, anterior cruciate ligament reconstruction.

**Table 6:** Chi-Square Results of Septic Arthritis Post-ACLR by Graft Type^*a*^

Graft Type	Total	Septic Arthritis Cases, n (%)
Allograft	368	0 (0)
Autograft hamstring tendon	3372	11 (0.33)
Autograft hamstring + allograft supplementation	86	0 (0)
Autograft patellar tendon	986	1 (0.10)
Autograft quadriceps tendon	187	1 (0.53)
Autograft iliotibial band	638	0 (0)
Degrees of freedom	5	
*P* value	.38	

a ACLR, anterior cruciate ligament reconstruction.

**Table 7:** Chi-Square Results of Septic Arthritis Post-ACLR by Age-Based Cohort^*a*^

Age-Based Category (Age Range, y)	Total	Septic Arthritis Cases, n (%)
Pediatric (<13)	606	0
Adolescent (13-19)	4123	12 (0.29)
Young adult (<19)	909	1 (0.11)
Degrees of freedom	2	
*P* value	.27	

a ACLR, anterior cruciate ligament reconstruction.

**Table 8:** Chi-Square Results of Septic Arthritis Post-ACLR by Presence of Concurrent Meniscal Surgery^*a*^

Meniscal Treatment	Total	Septic Arthritis Cases, n (%)	*P* Value
MM surgery	1250	2 (0.16)	.56
MM partial meniscectomy	398	0 (0)	>.99
MM repair	852	2 (0.23)	.98
LM surgery	2121	6 (0.28)	.52
LM partial meniscectomy	1048	3 (0.29)	.68
LM repair	1073	3 (0.28)	.71

a Meniscal surgery includes either partial meniscectomy or repair. ACLR, anterior cruciate ligament reconstruction; LM, lateral meniscus; MM, medial meniscus.

### Clinical Outcomes, Complications, and Subsequent Surgeries

While clinical outcomes were good—meaning successful eradication of infection with graft retention—for most patients, procedures subsequent to infection treatment were common, with 8 different patients (61.5%) undergoing a total of 14 different procedures at a median of 11 months (range, 3-62 months) after I&D. Six of those 8 patients had an ipsilateral meniscus tear (medial, lateral, or both) that had been treated at the index ACLR or subsequent arthroscopic procedure. One patient, who had originally undergone an ACLR with patellar tendon autograft, underwent 5 different knee arthroscopy procedures in the 15 months after I&D for infection, including lysis of adhesions, lateral retinacular release, patellar biopsy, and patellar chondroplasty for persistent peripatellar pain, and chondromalacia of the patellar cartilage. Eight of the 10 patients for whom septic arthritis was diagnosed in the first 6 weeks after the primary ACLR (not a subsequent, separate arthroscopic procedure closer to 12 months postoperatively) had adequately detailed return to sport (RTS), which was achieved at a median of 8 months (range, 6-12 months) after the primary ACLR. One of the 4 patients diagnosed with septic arthritis after a subsequent arthroscopic procedure, performed 8 months after primary ACLR, had adequately detailed RTS information, with RTS achieved at 11 months after ACLR. ACL graft retention was successful in all patients after primary treatment of the septic arthritis. However, 1 patient (7.7%) sustained an ACL graft rupture approximately 5 years after ACLR during a twisting injury while cutting during basketball. This patient underwent revision ACLR in 2019 with posterior tibialis allograft and was able to RTS 8.8 months after the revision procedure. Among the 8 patients (61.5%) who underwent 14 subsequent surgeries after septic arthritis treatment, 5 patients had 1 additional surgery, 2 patients had 2 additional surgeries, and 1 patient had 5 additional surgeries. In the overall septic arthritis population, the mean number of additional surgeries per patient is 1.08, or 0.75 if the outlier patient is excluded. The surgical details of the 8 patients with any post–septic arthritis treatment procedures are included in Table 9.

**Table 9:** Subsequent Surgeries After Irrigation and Debridement^*a*^

	Patient							
	A	B	C	D^*b*^	E^*b*^	F	G^*b*^	H^*b*^
Time from initial Sx, y	5.21	0.74	1.54	2.28	1.26	0.39	2.01	1.76
1st Sx diagnosis	ACL graft rupture, lateral meniscus tear	Arthrofibrosis, lateral meniscus tear	Medial meniscus tear (with bucket-handle displacement)	Medial meniscus tear, patellofemoral chondromalacia	Medial meniscus tear	Painful patella	Medial meniscus tear	Arthrofibrosis, medial + lateral meniscus tears
1st Sx procedure	Revision ACLR, partial lateral meniscectomy	AS LOA, MUA, partial lateral meniscectomy	AS medial meniscus repair	AS partial medial meniscectomy, chondroplasty	AS partial medial meniscectomy	Patellar biopsy	AS partial medial meniscectomy	AS LOA, MUA, medial + lateral painful medial lateral meniscectomies
Time from initial Sx, y				8.28	3.62	0.73		
2nd Sx diagnosis				Trochlear chondral flap chondromalacia	Loose body	Patellar chondrosis/fracture, lateral patellar tilt		
Time from initial Sx, y						0.77		
3rd Sx diagnosis						Diffuse synovitis (possible infection with elevated markers)		
3rd Sx procedure						Culture, debridement		
Time from initial Sx, y						1.09		
4th Sx diagnosis						Patellar chondromalacia		
4th Sx procedure						Partial debridement chondroplasty, patellar interosseous bone injection with ethanol for neurolysis		
Time from initial Sx, y						1.40		
5th Sx diagnosis						Chronic synovitis, knee and patellar pain		
5th Sx procedure						Patellar and lateral retinacular tissue biopsy, lateral retinacular release		

a ACL, anterior cruciate ligament; ACLR, anterior cruciate ligament reconstruction; AS, arthroscopic; LOA, lysis of adhesions; MUA, manipulation under anesthesia; RLB, removal of loose bodies; Sx, surgery.

b Underwent additional ipsilateral knee arthroscopy before diagnosis of septic arthritis.

## Discussion

Septic arthritis is a relatively rare complication after ACLR surgery, as evidenced by the 0.23% incidence in the 5638 cases investigated in the present study. It may be particularly rare in the pediatric population, as no such cases were identified in the 606 ACLRs performed in patients younger than 13 years of age. Treatment with I&D, graft retention, and antibiotic therapy represented a successful strategy in all patients in the present study.

The findings above suggest that the adolescent risk for post-ACLR septic arthritis may be similar to that of the adult or young adult population. In the present study, 0.30% of patients aged 13 to 19 years developed septic arthritis, while 0.11% of patients older than 19 years experienced this complication. These rates fall within the range of those previously published in the literature.^6,24,27,36,40^

Although previous publications have suggested an association of risk with graft type, because the vast majority of cases in the current investigation were performed with hamstring tendon autograft, it was not appropriately designed to reveal a similar finding or refute previous findings when studying this younger age group.^30,38^ Barker et al,^2^Özbek et al,^30^ and Schuster et al^38^ all found higher septic arthritis rates with use of hamstring tendon autografts than with use of other graft types. In the current study, quadriceps tendon autograft was actually found to have the highest incidence of septic arthritis (0.53%), but due to the low numbers of quadriceps tendon autograft surgeries performed in the overall cohort of ACLR surgeries, this analysis was not designed to achieve statistical significance.

Similar to previous studies, *S. aureus* and other *Staphylococcus* species remained the primary isolated organisms in this cohort.^18,36,40,44,45^ This may be of particular relevance with the increasingly widespread practice of intraoperative soaking of grafts in vancomycin before implantation, given the effectiveness of vancomycin to treat *Staphylococcus*.^‡^

Several studies have suggested that intraoperative graft soaking may significantly decrease the risk for septic arthritis.^§^ Most notably, the SANTI Study Group published their results in 5300 patients undergoing primary ACLR, finding a 5-fold greater risk of septic arthritis in patients who underwent treatment with a graft that had not been soaked in vancomycin during the procedure.^9^ Their meta-analysis results in the same publication demonstrated an odds ratio of 14.4 for those who did not receive grafts presoaked in vancomycin.^9^ With several studies now demonstrating both efficacy and safety—including alleviating concerns about potential negative effects on graft viability—this intervention has gained significant attention among orthopaedic surgeons and has recently been adopted at the study institution as well.^‖^ Prospective research efforts to assess the effectiveness of this intervention in preventing septic arthritis in the younger age groups treated at the study intervention are underway. However, the cohort in the current study was treated before the adoption of this intraoperative soaking practice.

Importantly, the current study suggests that treatment after the diagnosis of septic arthritis with aggressive early surgical I&D and extended antibiotics, for a mean of 6 weeks after I&D, represents a viable strategy. All 13 patients who developed septic arthritis were able to retain their ACL grafts, and only 1 patient ultimately required revision ACLR at 6 years after the index procedure. Successful treatment with graft retention is consistent with the findings of other studies on overall management strategies as well, several of which have also shown acceptable results in side-to-side laxity comparisons and subjective patient-based outcome scores.^¶^ Furthermore, Pogorzelski et al^31^ reported in a retrospective cohort study that their 21 patients treated with initial graft retention obtained significantly better subjective and objective outcomes than the 12 patients treated with initial graft removal. The findings of the current study, in combination with the literature, suggest that swift diagnosis with early debridement and antibiotics can be associated with attempted graft retention and may be the optimal strategy for the adolescent ACLR population as well. Given that this particular age group is the most commonly affected by ACL tears and has the highest rates of post-ACLR complications and graft rupture rates, addressing the complication of septic arthritis effectively is of critical importance.^28,33^

However, despite the effectiveness of surgical and antibiotic-based interventions for septic arthritis, post-I&D procedures were common, having been pursued in more than one-half of the study population. Revision ACLR rates after septic arthritis have been reported in the literature to range from 6.9% to 25%, with most large samples between 7% and 12%.^1,19,35^ These are comparable to the 7.7% revision rate found in the present sample. Rates of arthrofibrosis between 9.7% and 17% have been reported in the literature, which are also similar to the 15.4% rate found in the present sample.^22,48^ The rate of patients in the present sample who underwent additional procedures for meniscal or patellofemoral pathology (61.5%) appears to be relatively high, especially considering that some of these patients required multiple operations.

While few studies published to date have described the proportion of patients after ACLR septic arthritis who require further meniscal or cartilage procedures, Schollin-Borg et al^36^ reported that 20% of their patients required further meniscal surgery after ACLR septic arthritis, which is notably lower than the rate in the present study. McAllister et al^25^ has suggested that postinfectious articular cartilage and bone damage in patients who have septic arthritis after ACLR can lead to worse functional outcomes, which may explain the need for additional procedures for some of these patients. Lee et al^21^ suggested that cultured chondrocytes challenged with *S. aureus* bacteria demonstrated signs of apoptosis and continued elevated caspase activity, even after lavage and vancomycin treatment. The effects of septic arthritis on the integrity or health of the meniscus are not clearly established, but fibrocartilage may also be affected adversely, either as a direct effect of the intra-articular bacteria on the meniscus itself, or indirectly with the chronic inflammatory milieu of the synovial fluid containing activated enzymes. The current study’s high rate of 6 of 8 patients having an ipsilateral meniscus tear requiring arthroscopic treatment and 1 patient with patellar pain refractory to multiple interventions may be supportive of such destructive properties of active infection inside the knee joint, even with the young age of the patients in the current study cohort.

While the present study addresses a relative void in the literature by investigating results from a large single-institution database of patient populations who are otherwise underrepresented in the literature on post-ACLR septic arthritis, several study limitations warrant mention. The study is retrospectively designed and therefore subject to inherent biases, lack of control for potential confounders, and inconsistent reporting of symptoms and laboratory findings. The overall cohort of patients who developed septic arthritis may have been underreported by current methods, as some patients may have been evaluated at another institution in this highly mobile subpopulation. The data are furthermore not representative of the incidence after vancomycin graft soaking, as this was not yet part of the practice. Finally, the data may be inadequate to definitively identify any association of graft type with septic arthritis risk, as there were relatively few quadriceps grafts used during the study period, and hamstring grafts represented approximately 60% of the total sample used in the study period. Furthermore, while patellar tendon autografts did show a septic arthritis incidence of only 0.1% compared with 0.33% in the hamstring subset, this represented only a single case and therefore makes firm conclusions difficult to draw.

Despite these limitations, the primary finding that post-ACLR septic arthritis is exceptionally rare in the pediatric population, but is likely found at comparable rates in the adolescent population and young adult patients, is important for caregivers of younger ACLR patient populations, which are expanding more rapidly than the older age groups. Future work investigating the effect of vancomycin soaking on these findings in this age group, and related work on the effect of the growing practice of adjunctive lateral procedures, such as lateral extra-articular tenodesis and anterolateral ligament reconstruction, on the risk for developing post-ACLR septic arthritis, is critical to continue to elucidate all aspects of this impactful complication after a common surgery.

## Conclusion

Septic arthritis after ACLR is a rare complication, especially in pediatric patients. Adolescent patients appear to experience this complication at a similar rate to young adults. Most cases are caused by *S. aureus* or other *Staphylococcus* species, without a clear association with specific graft type or presence of concurrent meniscal surgery in the present study. Prompt diagnosis and treatment with graft retention, ≥1 arthroscopic surgical debridements, and adequate antibiotic therapy represent viable treatment strategies for these patients. The present study does not suggest an elevation in risk of ACL graft rupture requiring subsequent revision ACLR surgery, but subsequent other surgeries to address stiffness and meniscal and chondral pathology were not uncommon, occurring in just more than one-half of the study population.

## Footnotes

‡ References 3, 4, 9, 12, 16, 17, 20, 24, 29, 49.

§ References 3, 4, 9, 12, 16, 17, 20, 24, 29, 49.

‖ References 3, 4, 9, 12, 16, 17, 20, 24, 29, 49.

¶ References 1, 7, 15, 22, 23, 31, 34, 39, 47, 48.
